# Supplementary material for: Comprehensive and Rapid Quality Evaluation Method for the Ayurvedic Medicine Divya-Swasari-Vati Using Two Analytical Techniques: UPLC/QToF MS and HPLC–DAD
Source: Pharmaceuticals (Basel). 2021 Mar 27;14(4):297. doi: 10.3390/ph14040297 (PMC8067215; doi:10.3390/ph14040297)
Supplement: Supplementary file 1 [file pharmaceuticals-14-00297-s001.pdf]

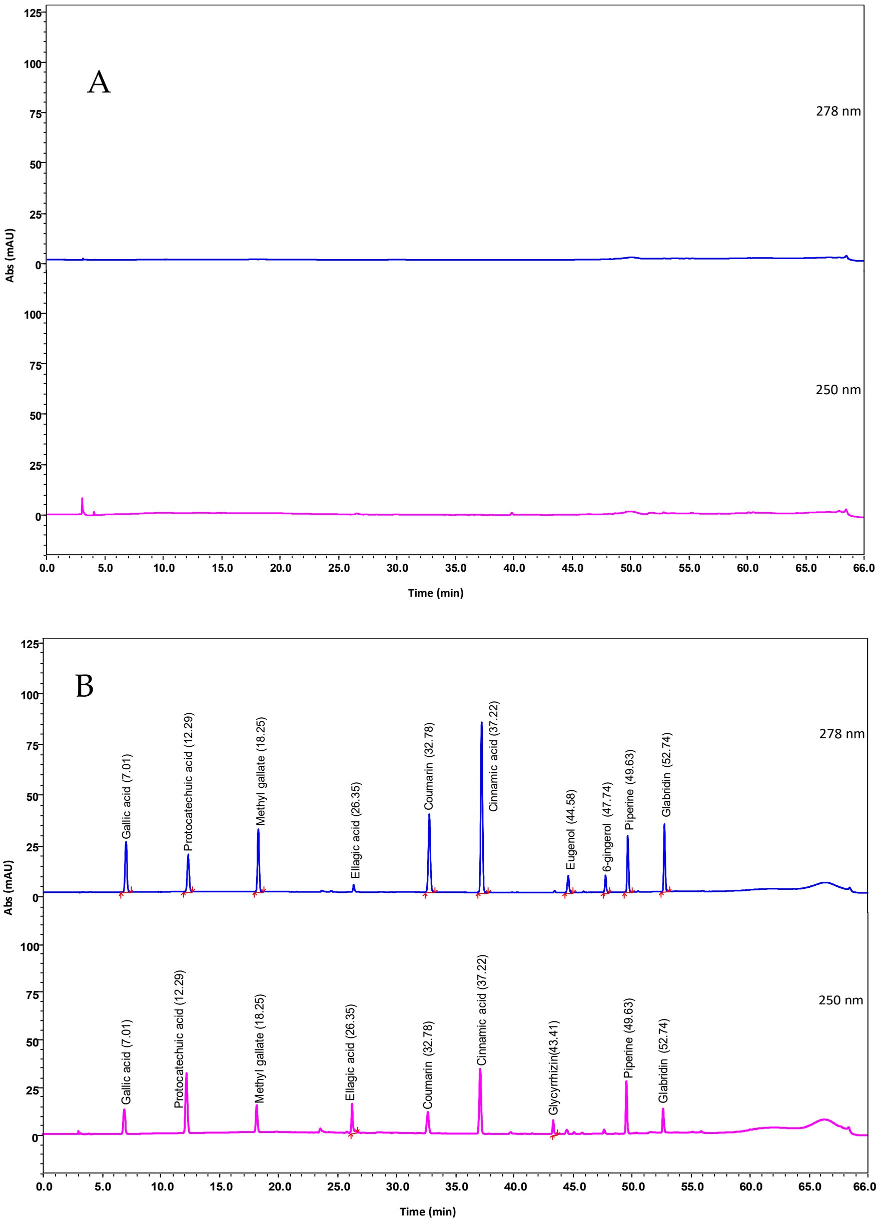

Figure.S1 (A) Chromatograph of blank solution at 278 nm (in blue) and 250 nm (in pink), (B) chromatograph of standard mixture solution at 278 nm (in blue) and 250 nm (in pink).

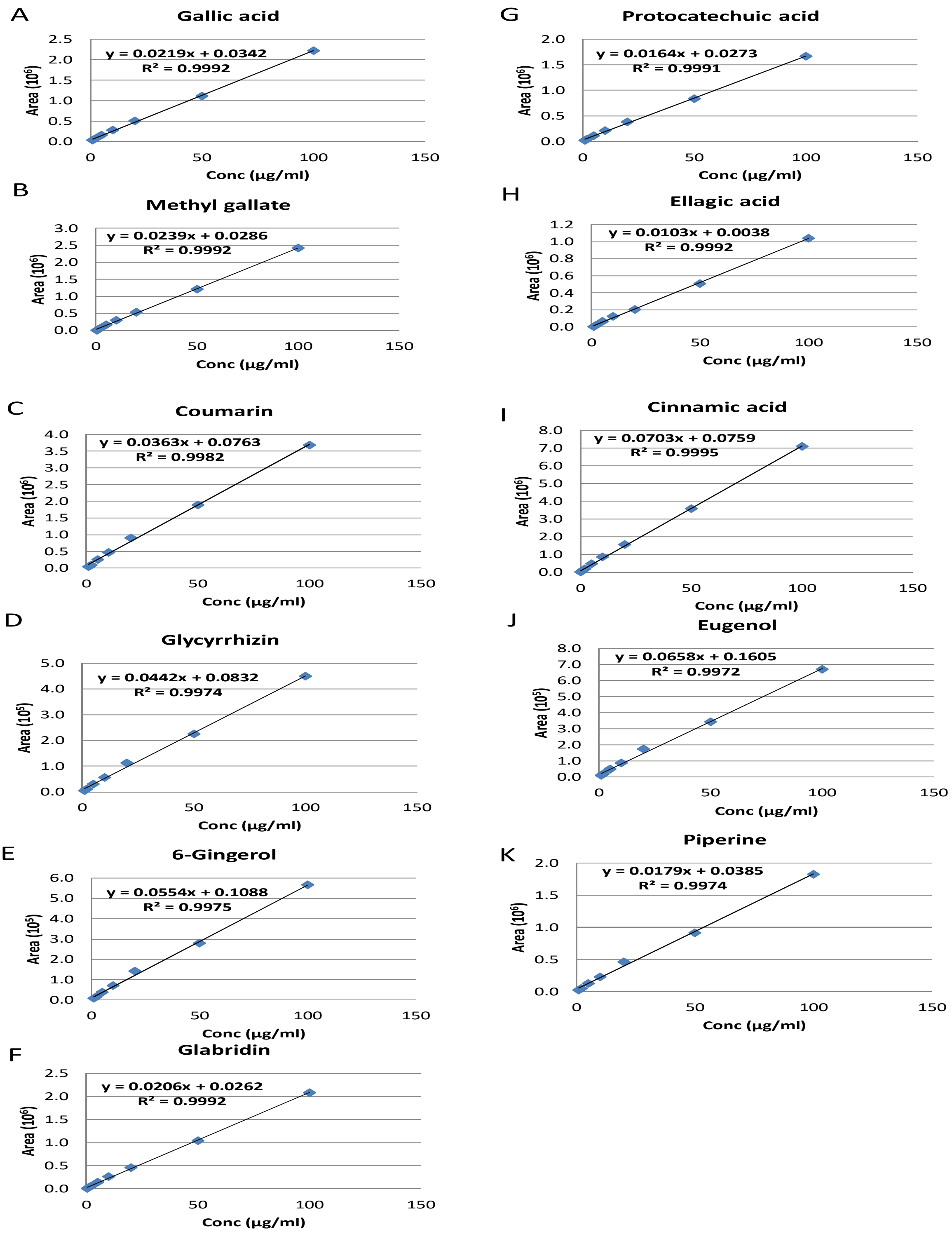

Figure.S2 Regression equation and correlation coefficient of A. gallic acid, B. methyl gallate, C. coumarin, D. glycyrrhizin , E. 6-gingerol, F. glabridin, G. protocatechuic acid, H. ellagic acid, I. cinnamic acid, J. eugenol, K. piperine

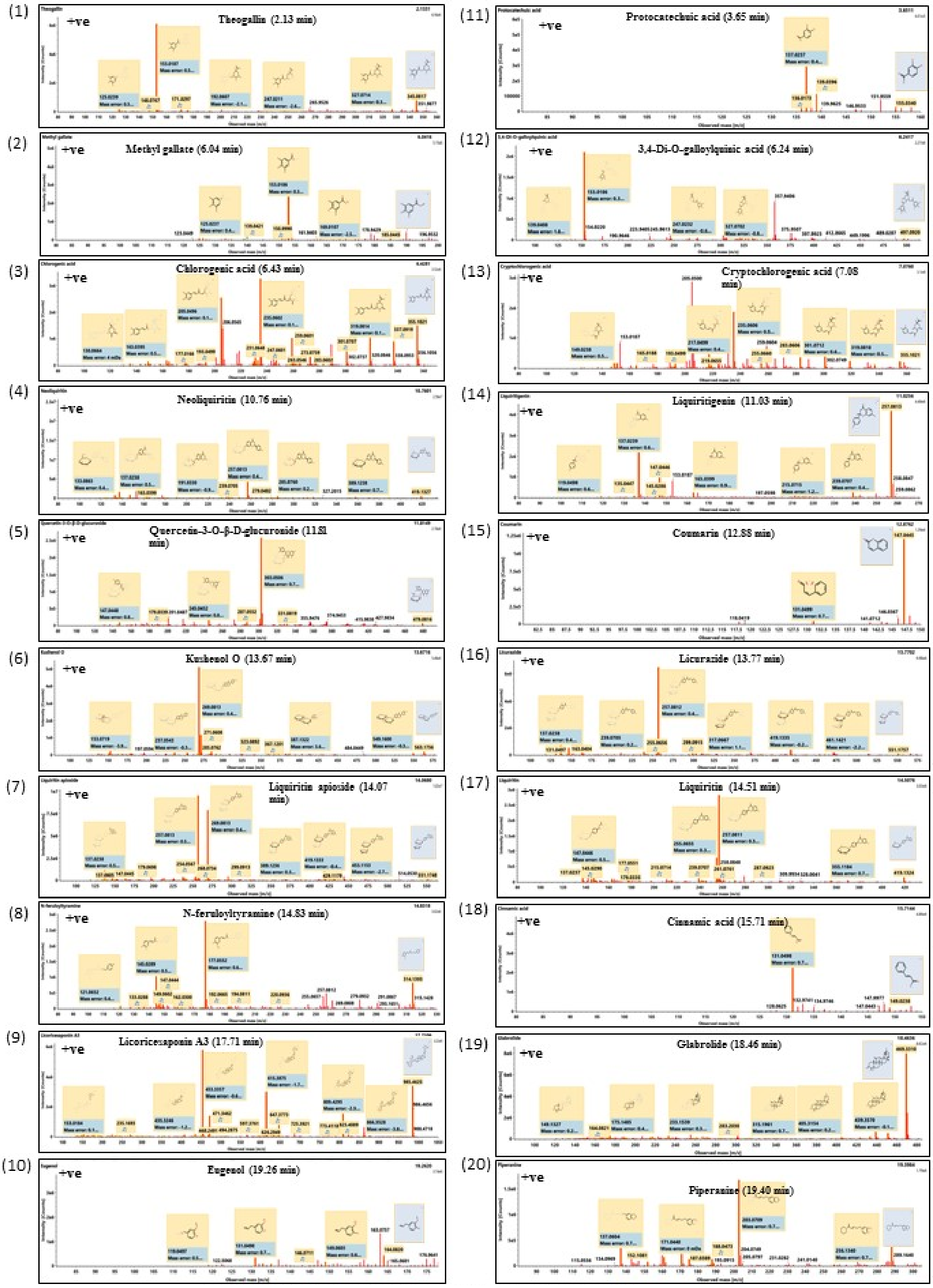
(1) +ve Theogallin (2.13 min)
(2) +ve Methyl gallate (6.04 min)
(3) +ve Chlorogenic acid (6.43 min)
(4) +ve Neoliquiritin (10.76 min)
(5) +ve Quercetin-3-O-β-D-glucuronide (11.81 min)
(6) +ve Kushenol O (13.67 min)
(7) +ve Liquiritin apioside (14.07 min)
(8) +ve N-feruloyltyramine (14.83 min)
(9) +ve Licoricesaponin A3 (17.71 min)
(10) +ve Eugenol (19.26 min)
(11) +ve Protocatechuic acid (3.65 min)
(12) +ve 3,4-Di-O-galloylquinic acid (6.24 min)
(13) +ve Cryptochlorogenic acid (7.08 min)
(14) +ve Liquiritigenin (11.03 min)
(15) +ve Coumarin (12.88 min)
(16) +ve Licuraside (13.77 min)
(17) +ve Liquiritin (14.51 min)
(18) +ve Cinnamic acid (15.71 min)
(19) +ve Glabrolide (18.46 min)
(20) +ve Piperanine (19.40 min)

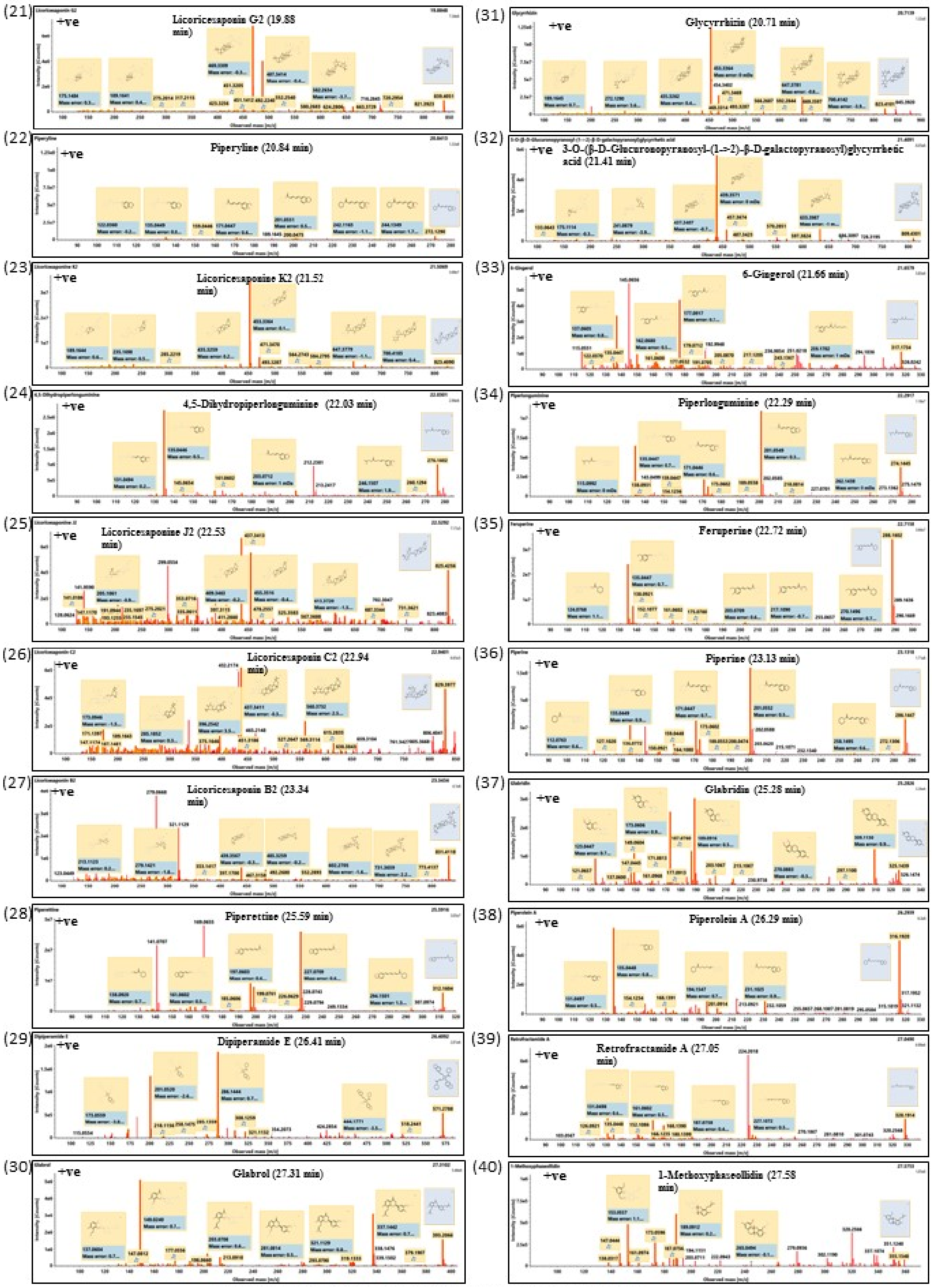
(21) +ve Licoricesaponin G2 (19.88 min)
(22) +ve Piperyline (20.84 min)
(23) +ve Licoricesaponine K2 (21.52 min)
(24) +ve 4,5-Dihydropiperlonguminine (22.03 min)
(25) +ve Licoricesaponine J2 (22.53 min)
(26) +ve Licoricesaponin C2 (22.94 min)
(27) +ve Licoricesaponin B2 (23.34 min)
(28) +ve Piperettine (25.59 min)
(29) +ve Dipiperamide E (26.41 min)
(30) +ve Glabrol (27.31 min)
(31) +ve Glycyrrhizin (20.71 min)
(32) +ve 3-O-(β-D-Glucuronopyranosyl-(1->2)-β-D-galactopyranosyl)glycyrrhetic acid (21.41 min)
(33) +ve 6-Gingerol (21.66 min)
(34) +ve Piperlonguminine (22.29 min)
(35) +ve Feruperine (22.72 min)
(36) +ve Piperine (23.13 min)
(37) +ve Glabridin (25.28 min)
(38) +ve Piperolein A (26.29 min)
(39) +ve Retrofractamide A (27.05 min)
(40) +ve 1-Methoxyphaseollidin (27.58 min)

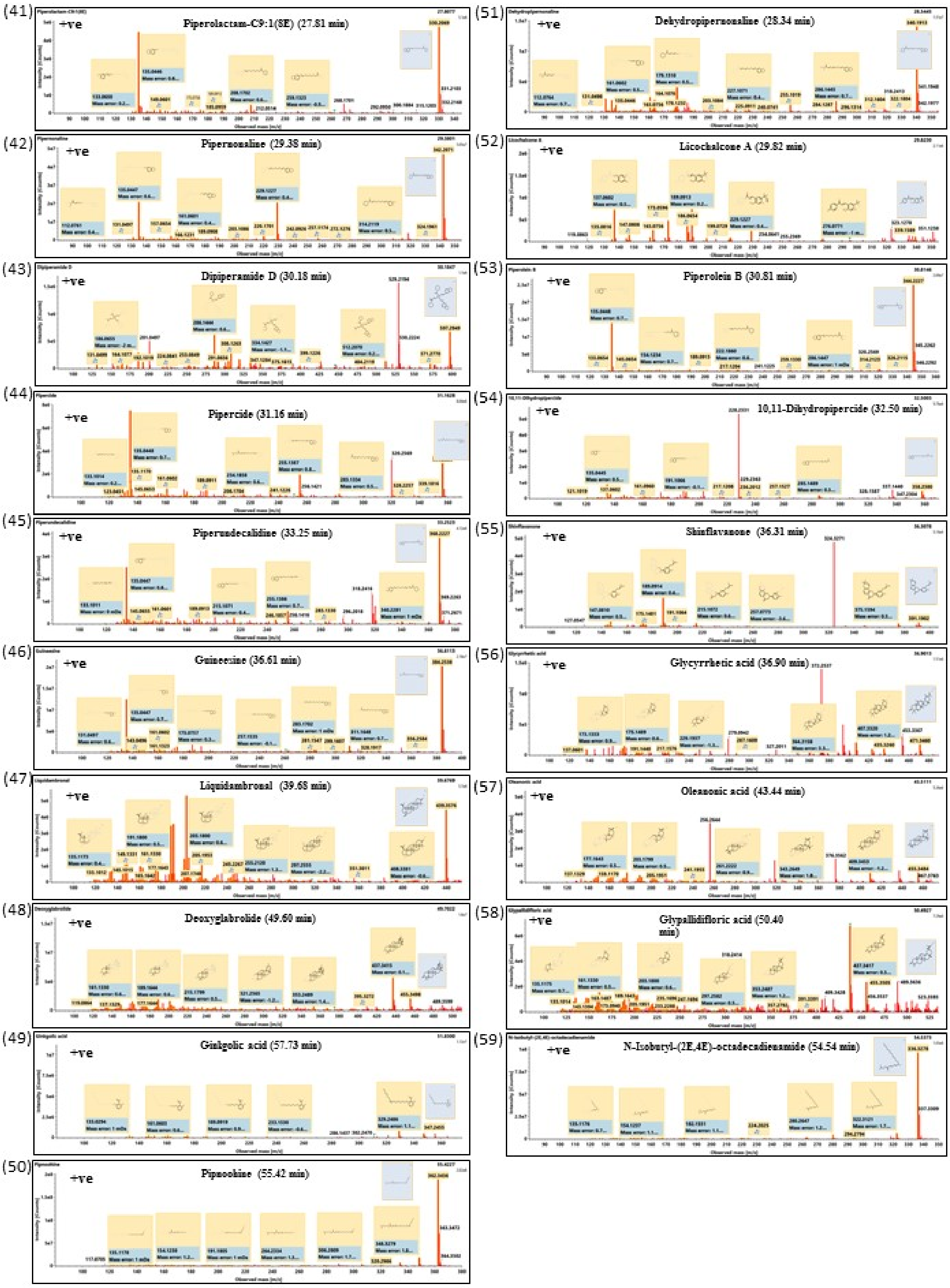

Figure. S3 Mass fragment pattern of the characterized fifty-nine compounds in Divya-swasari-vati (DSV) as observed in positive ionization mode. (Fig.3. A.), (Fig.3.B), (Fig.3. C.) Mass fragmentation of identified compounds in DSV (+ve mode). Where, (1) theogllin , (2) methyl gallate, (3) cholorogenic acid, (4), neoliquirtin, (5) quercetin-3-o-β-D-glucoronide, (6) kushenol O, (7) liquiritin apioside, (8) N-feruloyltyramine, (9) licoricesaponin A3, (10) eugenol, (11) protocatechuic acid, (12) 3, 4 Di-o-galloylquinic acid, (13) cryptochlorogenic acid, (14) liquiritigenin, (15) coumarin, (16) licurazide, (17) liquiritin, (18) cinnamic acid, (19) glabrolide, (20) piperanine, (21) licoricesaponin G2, (22), piperyline (23), licoricesaponin K2, (24) 4,5-Dihydropiperlonguminine, (25) licoricesaponin J2, (26) licoricesaponin C2, (27) licoricesaponin B2 (28) piperettine, (29) diperamide, (30) glabrol, (31) glycyrrhizin, (32) 3-o-(β-D glucoronopyranosyl (1-2)-β-D galacto pyranosyl) glycyrrhetic acid, (33) 6-gingerol, (34) piperlonguminine, (35) feruperine, (36) piperine, (37) glabridin,

(38) piperolein, (39) retrofractamide, (40) 1- methoxyphaseollidin, (41) piperolactam, (42) pipерononaline, (43) dipiperamide, (44) pipercide, (45) piperundecalidine, (46) guineesine, (47) liquidambronal, (48) deoxyglabrolide, (49) ginkgolic acid, (50) pipnoohine, (51) dehydropipernonaline,(52) licochalcone A (53) piperolein B, (54) 10,11- dihydropipercide, (55) shinflavanone, (56) glycyrrhetic acid, (57) oleanonic acid, (58) glypallidifloric acid, (59), N-isobutyl-(2E,4E)-octadecadienamide.

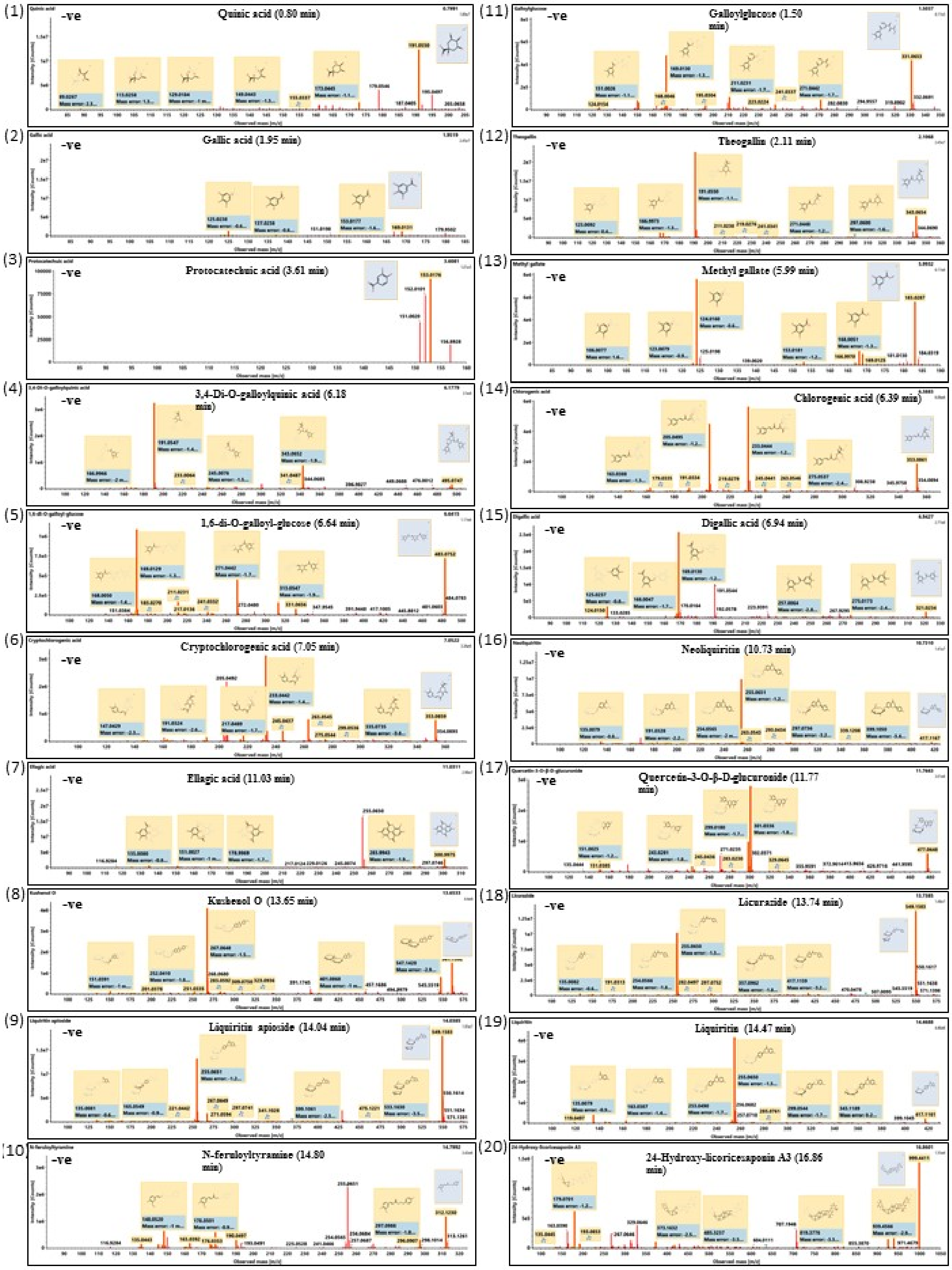
(1) -ve Quinic acid (0.80 min)
(2) -ve Gallic acid (1.95 min)
(3) -ve Protocatechuic acid (3.61 min)
(4) -ve 3,4-Di-O-galloylquinic acid (6.18 min)
(5) -ve 1,6-di-O-galloyl-glucose (6.64 min)
(6) -ve Cryptochlorogenic acid (7.05 min)
(7) -ve Ellagic acid (11.03 min)
(8) -ve Kushenol O (13.65 min)
(9) -ve Liquiritin apioside (14.04 min)
(10) -ve N-feruloyltyramine (14.80 min)
(11) -ve Galloylglucose (1.50 min)
(12) -ve Theogallin (2.11 min)
(13) -ve Methyl gallate (5.99 min)
(14) -ve Chlorogenic acid (6.39 min)
(15) -ve Digallic acid (6.94 min)
(16) -ve Neoliquiritin (10.73 min)
(17) -ve Quercetin-3-O-β-D-glucuronide (11.77 min)
(18) -ve Licuraside (13.74 min)
(19) -ve Liquiritin (14.47 min)
(20) -ve 24-Hydroxy-licoricesaponin A3 (16.86 min)

(21)
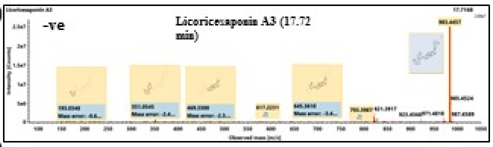

(22)
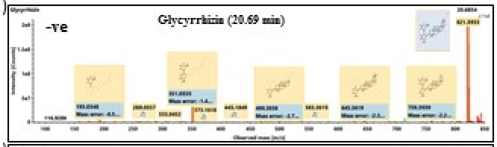

(23)
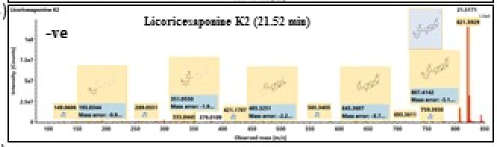

(24)
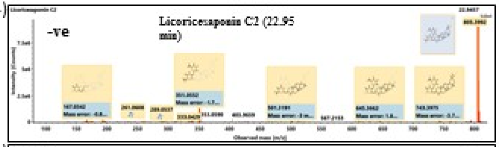

(25)
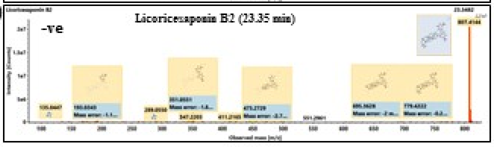

(26)
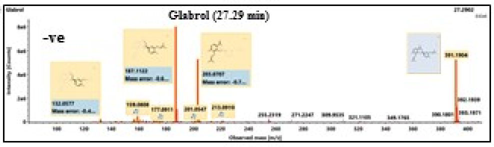

(27)
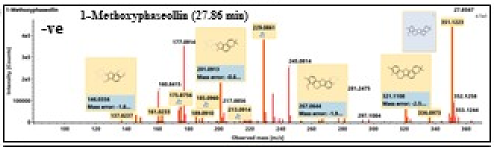

(28)
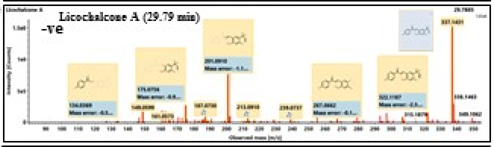

(29)
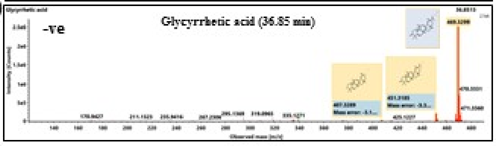

(30)
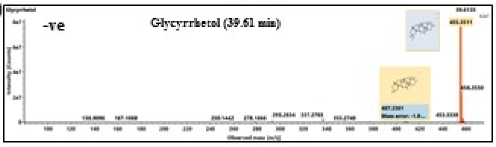

(31)
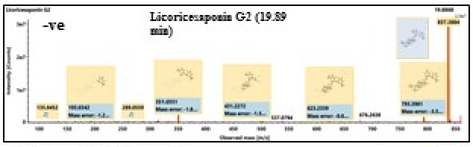

(32)
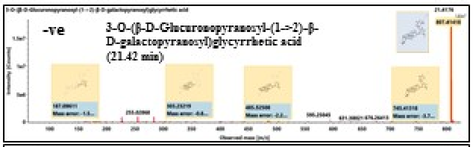

(33)
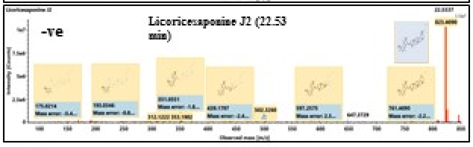

(34)
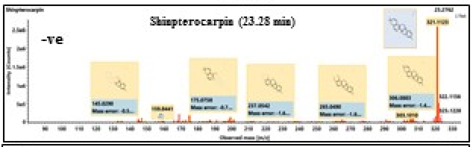

(35)
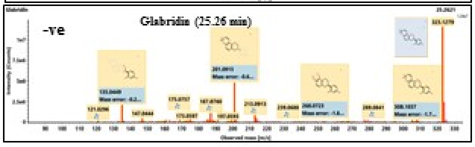

(36)
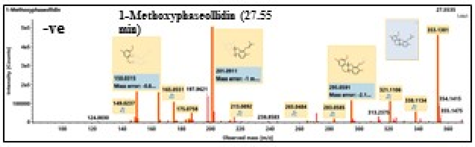

(37)
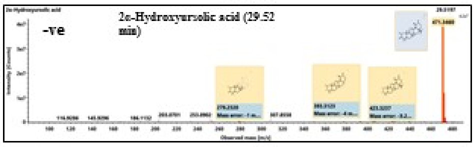

(38)
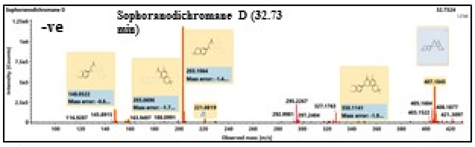

(39)
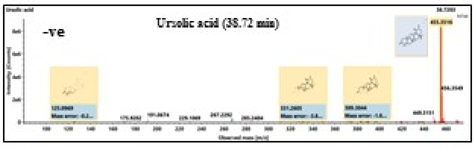

(40)
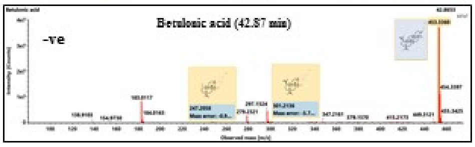

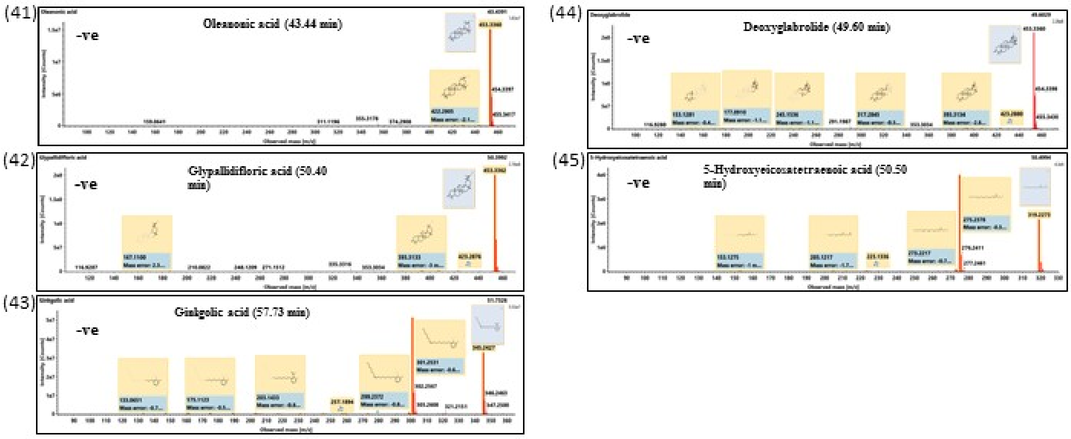

Figure. S4 Mass fragment pattern of the characterized forty-five compounds in Divya-swasari-vati (DSV) as observed in negative ionization mode (Fig.3. A.) (Fig.3. B.), (Fig.3. C.) Mass fragmentation of identified compounds in DSV (-ve mode). Where, (1) quinic acid , (2) gallic acid, (3) protocatechuic acid, (4), 3, 4 Di-o-galloylquinic acid, (5) 1,6- di-O-galloyl-glucose, (6) cryptochlorogenic acid, (7) ellagic acid, (8) kushenol O, (9) liquiritin apioside, (10) N- feruloyl tyramine, (11) galloylglucose, (12) theogallin, (13) methyl gallate, (14) cholrogenic acid, (15) digallic acid, (16) neoliquiritin, (17) quercetin-3-o-β-D-glucoronide, (18) licurazide, (19) liquiritin, (20) 24-hydroxy licoricesaponin A3, (21) licoricesaponin A3, (22) glycyrrhizin (23), licoricesaponin K2, (24) licoricesaponin C2, (25) licoricesaponin B2, (26) glabrol, (27) 1- Methoxyphaseollin (28) licochalcone, (29) glycyrrhetic acid, (30) glycyrrhetol, (31) licoricesaponin G2 (32) 3-o-(β-D glucoronopyranosyl (1-2)-β-D galacto pyranosyl) glycyrrhetic acid, (33) licoricesaponin J2, (34) shinterocarpine, (35) galbiridin, (36) 1-Methoxyphaseollidin, (37) 2-αhydroxyursolic acid, (38) sophoranodichromane D, (39) ursolic acid, (40) betulonic acid, (41) oleanonic acid, (42) glypallidifloric acid, (43) ginkgolic acid, (44) deoxyglabrolide, (45) 5-hydroxyeicosatetraenoic acid.

Table. S1 Residual sum of square (RSS) analysis of the targeted analytes.

Targeted phyto-metabolites	Residual sum of square (RSS)	Sum of square (SS)
**Gallic acid**	0.0030	3.8250
**Protocatechuic acid**	0.0020	2.1568
**Methyl gallate**	0.0038	4.9191
**Ellagic acid**	0.0007	0.8510
**Coumarin**	0.0186	10.5080
**Cinnamic acid**	0.0230	42.5767
**Glycyrrhizin**	0.0410	15.6086
**Eugenol**	0.0962	34.5957
**6-Gingerol**	0.0604	24.5318
**Piperine**	0.0067	2.5619
**Glabridin**	0.2779	365.1510
